# Chemo-immunotherapy improves long-term survival in a preclinical model of MMR-D-related cancer

**DOI:** 10.1186/s40425-018-0476-x

**Published:** 2019-01-10

**Authors:** Claudia Maletzki, Leonie Wiegele, Ingy Nassar, Jan Stenzel, Christian Junghanss

**Affiliations:** 1 Department of Medicine, Clinic III - Hematology, Oncology, Palliative Medicine, Rostock University Medical Center, Ernst-Heydemann-Str. 6, 18057 Rostock, Germany; 2 Core Facility Multimodal Small Animal Imaging, Rostock University Medical Center, Schillingallee 69a, 18057 Rostock, Germany

**Keywords:** Tumor lysate, Gemcitabine, MMR deficiency, In vivo imaging, Tumor microenvironment

## Abstract

**Background:**

Mismatch Repair Deficiency (MMR-D)-related tumors are highly immunogenic and constitute ideal vaccination targets. In a proof-of-concept study delayed tumorigenesis and prolonged survival has been shown in a clinically-relevant mouse model for MMR-D-related diseases (=MLH1 knock out mice). To refine this approach, vaccination was combined with immune modulatory low-dose chemotherapy to polarize immune regulatory subtypes.

**Methods:**

Mice (prophylactic: 8–10 weeks; therapeutic: > 36 weeks) received a single injection of cyclophosphamide (CPX, 120 mg/kg bw, i.p.) or gemcitabine (GEM, 100 mg/kg bw, i.p.) prior to vaccination (lysate of a gastrointestinal tumor allograft, 10 mg/kg bw, *n* = 9 mice/group). The vaccine was given repetitively (10 mg/kg bw, s.c., 4 x / once a week, followed by monthly boosts) until tumor formation or progression. Tumor growth ([^18^F] FDG PET/CT imaging) and immune responses were monitored (flow cytometry, IFNγ ELISpot). The microenvironment was analyzed by immunofluorescence.

**Results:**

Prophylactic application of GEM + lysate delayed tumorigenesis compared to lysate monotherapy and CPX-pre-treatment (median time of onset: 53 vs. 47 vs. 48 weeks). 33% of mice even remained tumor-free until the experimental endpoint (= 65 weeks). This was accompanied by long-term effect on cytokine plasma levels; splenic myeloid derived suppressor cells (MDSC) as well as regulatory T cell numbers. Assessment of tumor microenvironment from GEM + lysate treated mice revealed low numbers of MDSCs, but enhanced T cell infiltration, in some cases co-expressing PD-L1. Therapeutic chemo-immunotherapy (GEM + lysate) had minor impact on overall survival (median time: 12 (GEM + lysate) vs. 11.5 (lysate) vs. 3 weeks (control)), but induced complete remission in one case. Dendritic and T cell infiltrates increased in both treatment groups. Reactive T cells specifically recognized MLH1^−/−^ tumor cells in IFNγ ELISpot, but lacked response towards NK cell targets YAC-1.

**Conclusions:**

Combined chemo-immunotherapy impairs tumor onset and growth likely attributable to modulation of immune responses. Depleting or ‘re-educating’ immunosuppressive cell types, such as MDSC, may help moving a step closer to combat cancer.

## Background

Cancer vaccines (autologous, peptide-based, viral vector, and dendritic cells) provide an excellent tool to restore or augment antitumoral immune responses. Such vaccines exhibit unique tumor cell specificity and the potential to persuade durable, long-lasting efficacy because of T-cell driven immunologic memory induction [1]. They can be either given in a preventive or therapeutic setting [2]. Since the introduction of the first personalized prostate cancer vaccine in 2010 (= sipuleucel-T), many other immunotherapeutic approaches, such as adoptive cell transfer and oncolytic viruses were developed and some of them are still under clinical investigation [2, 3]. Restoration of immune responses with immune-checkpoint inhibitors such as anti-PD-1, anti-CTLA-4 and anti-PD-L1 is currently widely applied in the clinic and has shown remarkable success in the management of certain types of cancers [4–7]. However, recent studies describe intrinsic as well as acquired resistance mechanisms. Cancer vaccines may thus have a broader range of applications, not confined to a particular patient cohort and apart from this, with a better safety profile [8, 9]. Neoadjuvant anti-tumor vaccination was even shown to improve post-surgical survival in an experimental mouse model [10].

Inactivation of the mismatch repair (MMR) system defines a molecular subtype with great potential to be targeted immunologically. The molecular fingerprint of resulting tumors is microsatellite instability (MSI) characterized by an outstandingly high mutation burden and an accordingly high abundance of frameshifted neo-epitopes on the tumor cells’ surface [5, 11–13]. These neo-epitopes are unique to each individual patient, foreign to the immune system and represent ideal vaccination targets, without conferring risk to induce autoimmunity. Mutation-derived neoantigen cancer vaccines consequently entered clinical phases I/II (clinical trials.gov identifier: NCT01461148 & NCT01885702).

Additionally to choosing the right target antigen(s), modulating the tumor microenvironment is crucial to counteract immune evasion [14]. Tumors promote infiltration of regulatory T cells (Treg), tumor-associated macrophages, and myeloid-derived suppressor cells (MDSCs). These cellular subtypes are considered to thwart the innate (dendritic cells (DC) and NK cells) and adaptive (CD8^+^ T cells) arm of tumor immunosurveillance by secreting nitric oxide, reactive oxygen species and immunosuppressive cytokines such as IL-10 and TGF-β [15]. They even contribute to resistance towards immune checkpoint inhibition [16]. Neutralizing the effect of immunosuppressive subpopulations yet preserving T-cell function seems thus reasonable to increase vaccine efficacy.

In a previous study, delayed tumorigenesis and prolonged survival after repetitive application of a cancer vaccine was described in a clinically-relevant mouse model for MMR-D-related diseases [17]. To refine this approach, vaccination was combined with single low-dose chemotherapy as precondition to polarize immunosuppressive cells and thus modulate the immune system. Pretreatment was either done with Cyclophosphamide (CPX) or Gemcitabine (GEM) followed by repetitive vaccination of MLH1^−/−^ mice. Applying this concept we aimed at restoration of immune responses to break intrinsic tolerance against self neo-antigens.

## Material and methods

### Cell culture & drug response analysis

The MLH1^−/−^ cell lines were either established from gastrointestinal (cell lines: A7450 T1 M1 and 328 [18]) or lymphoid (cell line: 1351, origin: spleen; non-Hodgkin’s lymphoma (CD4^+^CD8^+^T-cell type) tumors. All cell lines were characterized (growth kinetic, phenotype) and successfully maintained in cell culture for > 30 passages. For in vitro drug response analyses, passages between 15 and 20 were used. Cells were cultured in DMEM/Ham’s F12 (Biochrom, Berlin, Germany) supplemented with 10% FCS (PAN-Biotech, Aidenbach, Germany) and 2 mM L-Glutamine (Biochrom) at 37 °C, 5% CO_2_. Cells were treated with increasing doses of standard chemotherapeutics GEM, CPX, 5-Flourouracil (5-FU), and Cisplatin for two cycles of each 72 h. Control cells were added medium only. Biomass quantification was done after staining residual epithelial cells with 0.2% crystal violet and subsequent measurement at 570 nm (Roth, Karlsruhe, Germany). Lymphoma cells’ viability was calculated after Calcein AM staining and subsequent fluorescence measurement at a wavelength of 485 nm (Glomaxx, Promega, Mannheim, Germany) [19]. In some experiments, phenotypic changes (determined as total amounts of target antigens after cell membrane permeabilization) as well as levels of immunogenic cell death (ICD) were determined upon 24 h treatment at doses corresponding to IC_30_ values. Supernatants were collected and amounts of high-mobility group protein 1 (HMGB1) were measurement by ELISA according to the manufacturers’ instructions (Abbexa, Cambridge, UK). Levels of surface bound Calreticulin (CalR, Biozol, Eching, Germany) as additional ICD marker were determined by flow cytometry and confocal laser scanning microscopy, respectively (Zeiss, Jena, Germany) using 20x objectives. In this analysis, CalR positivity (green fluorescence) was scored in 5 high-power fields, with each field having at least 50 DAPI stained cells (blue fluorescence).

### MLH1^−/−^ mouse model

Homozygous mice were obtained by breeding heterozygous males and females of the ≥F5 generation, originally obtained from the NCI mouse repository. All animals received standard laboratory chow and free access to water. Mice were bred in the animal facilities (University of Rostock) under specified pathogen-free conditions. Trials were performed in accordance with the German legislation on protection of animals and the Guide for the Care and Use of Laboratory Animals (Institute of Laboratory Animal Resources, National Research Council; NIH Guide, vol.25, no.28, 1996; approval number: LALLF M-V/TSD/7221.3–1.1-053/12–1 and 026/17). MLH1 genotyping was done according to [20].

### Vaccine preparation & in vivo vaccination protocol

Vaccine preparation was done as previously described [17]. Briefly, outgrowing allografts were lysed using repetitive freeze/thaw cycles (*n* = 4). Protein lysates were gamma irradiated (60 Gy) and frozen immediately in aliquots at − 80 °C before in vivo application.

Prophylactic approach: Mice (8–10 weeks old) received a single injection of CPX (120 mg/kg bw, i.p., *n* = 9 mice) or GEM (100 mg/kg bw, i.p., n = 9 mice) prior to vaccination (− 24 h). Thereafter, the vaccine was given repetitively by subcutaneous injections (10 mg/kg bw, s.c., qw 1–4). Control mice were only given GEM or CPX (*n* = 4 mice per group) in equivalent doses. Vaccination was continued until tumor development (monthly injections: 2.5 mg/kg bw).

Therapeutic approach: Mice who had gastrointestinal tumors (GIT) confirmed by small animal ^18^F-FDG PET/CT imaging [17] were treated by chemo-immunotherapy. Mice received a single injection of GEM (− 24 h, 100 mg/kg bw, i.p., *n* = 5 mice) followed by four weekly injections of the vaccine (10 mg/kg bw, s.c.) in the first phase. Vaccination was continued (2.5 mg/kg bw, biweekly) until tumors progressed. PET/CT imaging was repeated on days 28 or 35 of therapy. Control mice received single injections of GEM (100 mg/kg bw, i.p., *n* = 3 mice) or vaccine (10 mg/kg bw, s.c., 4 x / once a week, followed by biweekly injections at 2.5 mg/kg bw). Blood samples were taken before treatment (prophylactic and therapeutic) and regularly during the experiment (day 28, 56, and 84). Tumor-bearing mice were sacrificed. Blood samples, tumors and spleens were taken from all animals for further analysis.

### PET/CT imaging

PET/CT imaging scans were performed on a small animal PET/CT scanner (Inveon PET/CT, Siemens Medical Solutions, Knoxville, TN, USA) according a standard protocol. Mice with suspected GIT (*N* = 5/per group) were anaesthetized using isoflurane (4% for induction and 1–2,5% maintenance during preparation and scanning) and were injected intravenously with a mean dose of 17.12 ± 1.81 MBq [^18^F] FDG via a custom-made micro catheter placed in the tail vein. After an uptake period of 60 min, mice were imaged in prone position for 15 min as described [17]. Throughout the imaging session, respiration of the mice was controlled and body temperature was constantly kept of 38 °C via a heading pad. The PET image reconstruction method consisted of a 2-dimensional ordered subset expectation maximization algorithm (2D-OSEM) with four iterations and 6 subsets. Attenuation correction was performed on the basis whole body CT scan and a decay correction for [^18^F] was applied. PET images were also corrected for random coincidences, dead time and scatter. Tumor volumes and SUVs were determined using Inveon Research Workplace 4.2 software.

### Flow cytometric phenotyping

Blood samples were taken routinely from the retrobulbar venous plexus of vaccinated and control mice. Blood samples were stained with a panel of conjugated monoclonal antibodies (mAb, 1 μg each) followed by lysis of erythrocytes (155 mM NH_4_Cl (MERCK Millipore, Darmstadt, Germany), 10 mM KHCO_3_ (MERCK Millipore), 0.1 mM EDTA (Applichem, Darmstadt, Germany). Intracellular staining was done upon incubation with 1x Intracellular Staining Perm Wash Buffer (Biolegend, Koblenz, Germany). Negative controls consisted of lymphocytes stained with the appropriate isotypes (Biolegend). Cultured tumor cell lines were phenotyped with FITC-, PE-, APC-, PE-Cy7-, or APC-Cy7-labeled mAbs as follows: PD1, PD-L1, CTLA-4, LAG-3, TIM-3, IDO-1, IFN-γ, and TNF-α (Biolegend). Cells were washed, resuspended in PBS and analyzed by flow cytometry on a FACS Verse Cytometer (BD Pharmingen). Data analyses were performed using BD FACSuite software (BD Pharmingen).

### Immunofluorescence

Cryostat sections of 4 μm were air-dried and fixed in cold pure methanol for 8 min. Unspecific binding sites were blocked in 2% BSA (Roth) for 2 h followed by incubation with 1 μg of the following FITC- and PE-labeled mAbs: CD4, CD8α, CD11b, CD19, CD20, Gr1 (Immunotools, Friesoythe, Germany), CD11c, CD104, LAG-3, PD-1, NK1.1, F4/80, and PD-L1 (Biolegend). Sections were washed and embedded in Roti Mount Flour Care DAPI to stain nuclei (Roth, Karlsruhe). Visualization of target genes was done on a confocal laser scanning microscope (Zeiss, Jena, Germany) using 20x objectives.

### IFNγ–ELISpot assay

Functional immunological in vitro assays included IFNγ ELISpot and a flow cytometric cytotoxicity assay. All procedures were done as described [17]. Briefly, MLH1^−/−^ target cells were co-cultured with peripheral blood leukocytes or splenocytes (1 × 10^4^/well) were added to targets in triplicates and overnight. Upon visualization, pots were counted using an ELISpot reader. Presented are the numbers of IFN*γ*–secreting cells per 10,000 effector cells corrected for background levels (no target cells and no effector cells, usually ≤5 spots/well).

### Procartaplex cytokine assay

A panel of plasma cytokine levels from treated and control mice were determined according to the manufacturer’s instructions of the Procartaplex™ multiplex immunoassay. Measurement as well as cytokine quantification was done on a Bioplex 2000 (Bio-Rad Laboratories GmbH, Munich, Germany) in combination with the Bio-Plex Manager Software.

### Statistics

All values are expressed as mean ± SD. Differences between individual treatment schedules in vitro were examined by applying one way ANOVA (Holm Sidak method). After proving the assumption of normality (Kolmogorov-Smirnov test), differences between treated and control mice were determined using the unpaired Student’s *t*-test. Kaplan-Meier survival analysis was done by applying log rank test. The tests were performed by using Sigma-Stat 3.0 (Jandel Corp, San Rafael, CA). The criterion for significance was set to *p* < 0.05.

## Results

### In vitro drug selection for chemo-immunotherapy

**Fig. 1:**
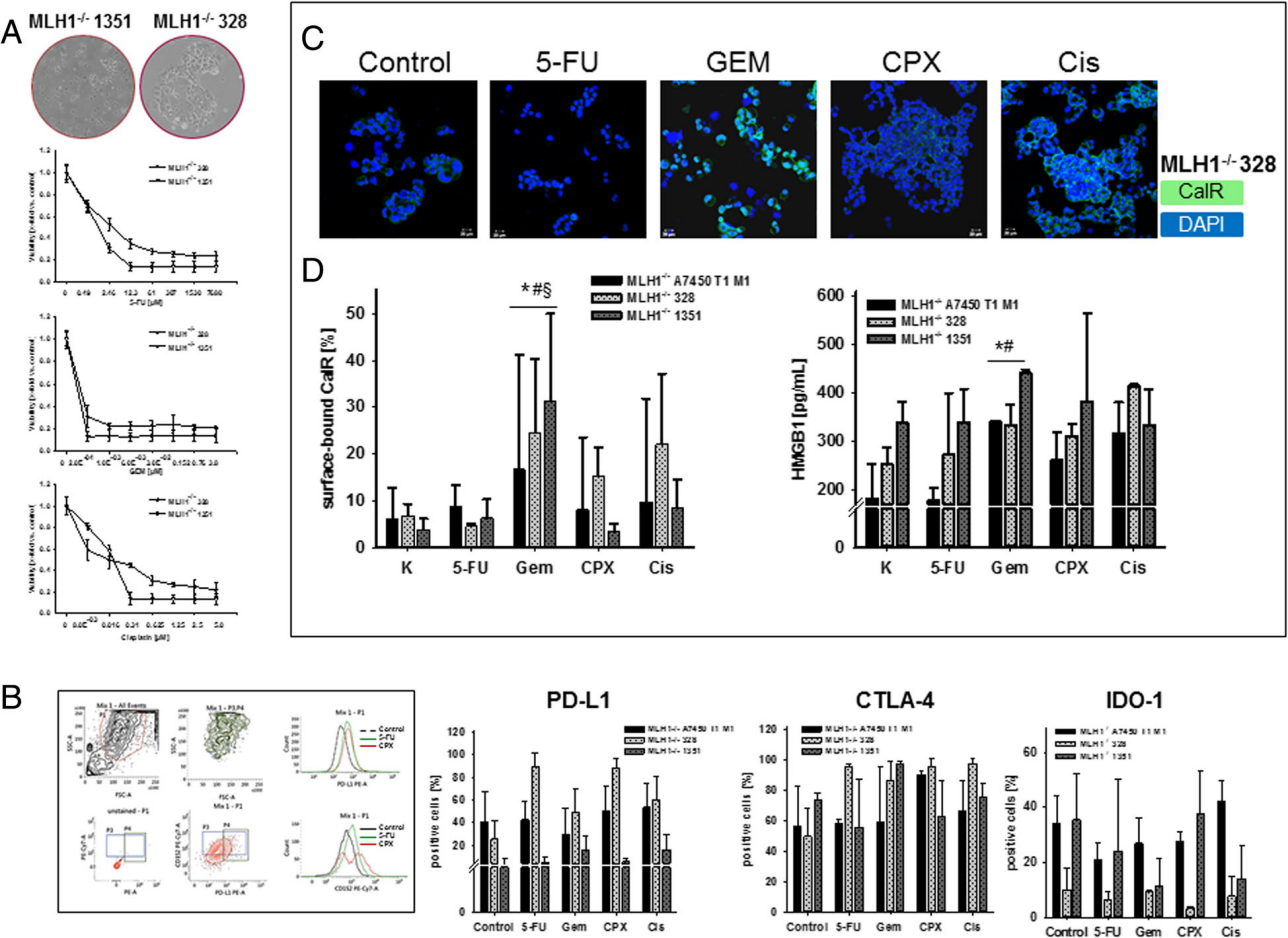
Drug response, immunophenotyping and ICD detection in MLH1^−/−^ cell lines A7450 T1 M1, 328, and 1351. **a** Dose response curves of MLH1^−/−^ 328 und MLH1^−/−^ 1351 cells showing dose-dependent reduction of cell viability. All cell lines were resistant towards CPX (*data not shown*). Data from MLH1^−/−^ A 7450 T1 M1 cells are taken from [18]. **b** Flow cytometric gating strategy and quantitative phenotyping. Cells were treated with chemotherapeutic drugs for 24 h treatment, followed by cell harvest and staining as stated in material and methods. Amounts of cell-surface bound and intracellular molecules were analyzed by multi-color flow cytometry. Results are given as percentage number of cells (%) within 20,000 cells ± standard deviation of three independent experiments each performed in triplicates. **c** Representative confocal laser scanning microscopy images of MLH1^−/−^ 328 cells showing increased CalR exposure on the tumor cells’ surface. **d** Flow cytometric quantification of surface-bound CalR (left graph) as well as quantification of HMGB1 (right graph) in supernatants of MLH1^−/−^ tumor cells after treatment with different chemotherapeutic drugs. Control cells were left untreated. Experiments were repeated three times each of them performed in duplicates. Values of are given as mean ± SD. *p < 0.05 vs. control; # p < 0.05 vs. 5-FU; § p < 0.05 vs. CPX; one-way ANOVA (Holm Sidak method)

In a preliminary in vitro screening, MLH1^−/−^ cell lines were exposed to increasing drug concentrations (representative dose response curves of 2/3 cell lines are shown in Fig. 1a). All cell lines showed high responsiveness towards GEM, with IC_50_ levels below 0.1 μM (Table 1). A more individual response pattern was seen after Cisplatin and 5-FU treatment, with MLH1^−/−^ 1351 lymphoma cells being more vulnerable towards 5-FU than the epithelial cells (Table 1). In line with their molecular signature, the tested cell lines were completely resistant towards CPX, even after two cycles at a dose of > 300 μM. Abundance of immune-checkpoint molecules was quite heterogeneous between cells (Fig. 1b). GEM and 5-FU reduced amounts of intracellular IDO-1 in 2/3 cases (Fig. 1b). The altered immune phenotype was accompanied by increased levels of surface bound CalR (Fig. 1c) as well as elevated HMGB1 secretion (Fig. 1d) indicative for induction of ICD [21]. Effects were most prominent after GEM exposure, while CPX and 5-FU had minor influence on these markers.

**Table 1:** In vitro drug response of MLH1^−/−^ cell lines

Drug	IC_50_ value [μM]		
	MLH1^−/−^ A7450 T1 M1^a^	MLH1^−/−^ 328	MLH1^−/−^ 1351
GEM	0.05	0.07	< 0.02
Cisplatin	0.63	0.05	0.06
5-FU	2.15	3.57	1.00
CPX	> 300	> 300	> 300

*GEM* Gemcitabin, *5-FU* 5-Fluorouracil, *CPX* Cyclophosphamide; ^a^ [18]

To examine the effect of GEM and CPX on normal cells’ viability, leukocytes were cultured in the presence of different low concentrations for 24 h and 48 h, respectively (*data not shown*). No cytotoxicity was observed providing a ready basis for subsequent in vivo preconditioning.

### Chemoprevention delays tumorigenesis by long-term immune modulation

**Fig. 2:**
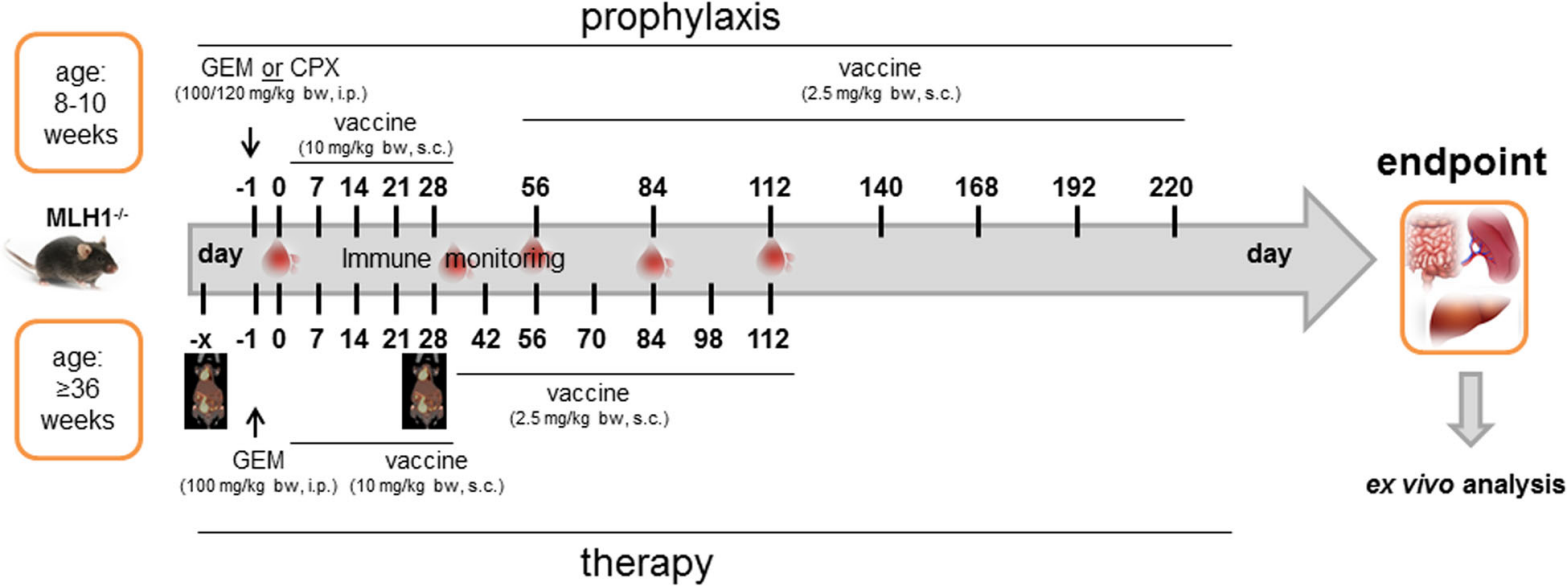
Treatment scheme. Mice received prophylactic or therapeutic applications of the vaccine, chemotherapy or a combination of both. The vaccine was prepared from an MLH1^−/−^-derived allograft. Immune monitoring as well as assessment of general behavior was done routinely during vaccination and at the experimental endpoint. Prior to therapy, mice underwent in vivo PET/CT imaging to confirm gastrointestinal tumorigenesis. Ex vivo analyses were done as described

Above data hint towards strong cytotoxic activity of selected drugs against MLH1^−/−^ − target cells without impairing normal cells’ viability. We therefore examined the immune modulatory activity of chemotherapeutics when given before (= prophylactic approach) and after tumor establishment (= therapeutic approach) in vivo. The agents GEM and CPX were selected on the basis of supposed immunomodulatory activities such as reducing MDSC numbers in vivo and augmenting antigen-specific cellular antitumor immunity to promote T helper immunity [22]. Chemotherapeutics were given once before vaccination at doses of 100 and 120 mg/kg bw, respectively, to boost tumor immunity. Control mice were given single injection of either substance at given doses. A detailed treatment schedule is given in Fig. 2.

**Fig. 3:**
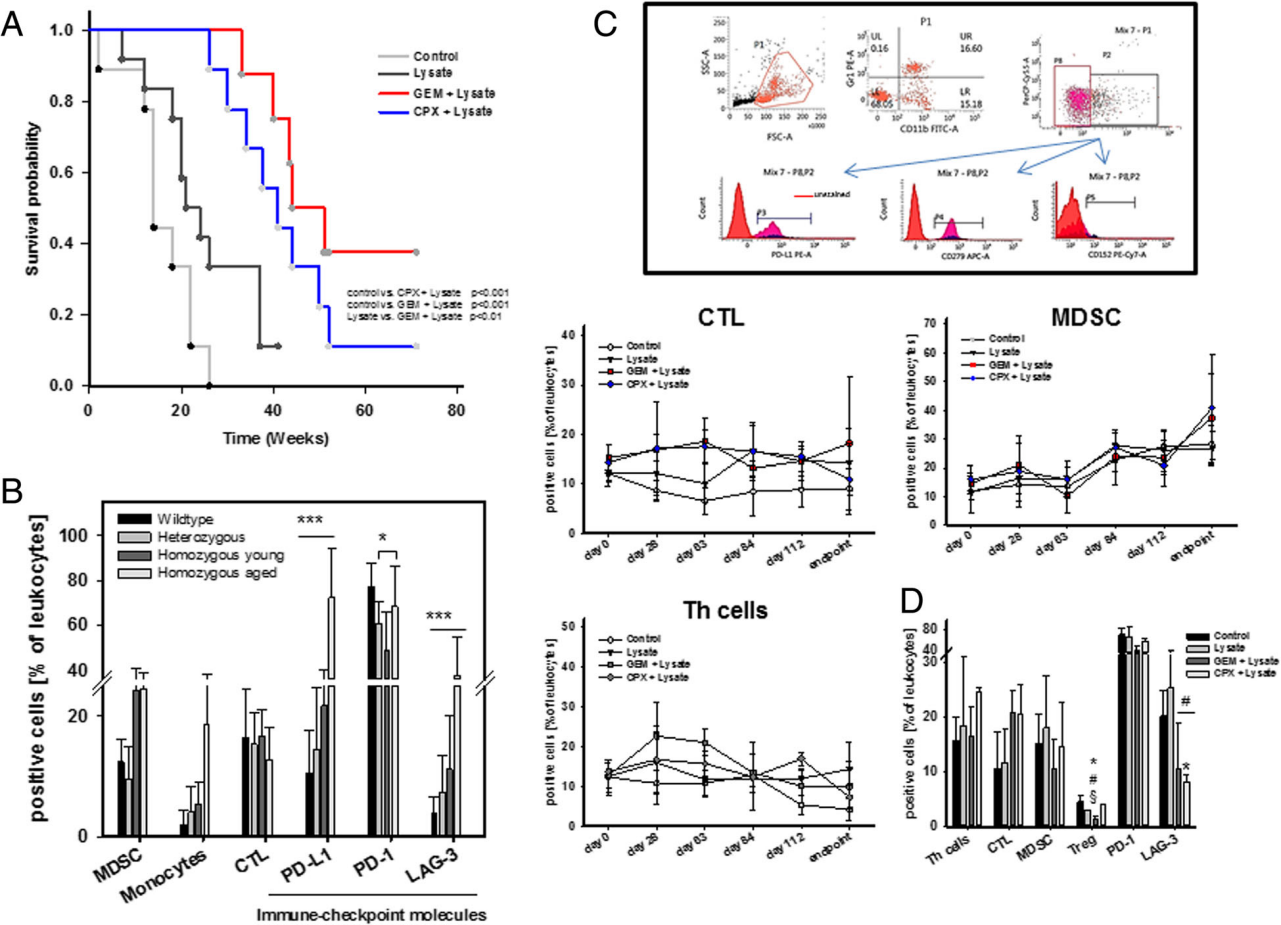
Kaplan-Meier survival curve and immune monitoring during prophylactic chemo-immunotherapy. **a** Log rank survival analysis of treated and control mice. Mice receiving chemo-immunotherapy were given GEM (100 mg/kg bw, i.p., *n* = 9 mice) or CPX (120 mg/kg bw, i.p., *n* = 9 mice) 24 h before vaccination (tumor lysate), followed by repetitive local applications of the vaccine (10 mg/kg bw, s.c.). An additional group of mice received the vaccine (10 mg/kg bw, s.c., *n* = 6 mice). Control mice received single GEM (100 mg/kg bw, i.p.) or CPX (120 mg/kg bw, i.p., *n* = 4 mice per group) injections or were left untreated. *p* < 0.001 control vs. CPX + lysate; p < 0.001 control vs. GEM + lysate; *p* < 0.01 lysate vs. GEM + lysate. **b**, **c** Flow cytometric immune monitoring of blood leukocytes. Presented data refer to % numbers of viable immune cells, by excluding dead cells and debris. **b** Immune cell distribution among wildtype (*n* = 4, aged: 15–32 weeks), heterozygous (*n* = 7, aged: 15–32 weeks) as well as young (*n* = 5, ≤20 weeks) and aged MLH1 knockout mice (*n* = 10, ≥32 weeks). *** *p* < 0.001 homozygous aged vs. wildtype, heterozygous and homozygous young. *p < 0.05 wildtype vs. homozygous young; one-way ANOVA (Holm Sidak method). MDSC – CD11b^+^Gr1^+^ myeloid-derived suppressor cells; Monocytes – CD200R^+^ (**c**) Immune status was determined routinely during vaccination and at the experimental endpoint. Upper panel: representative FACS plots showing gating strategy for assessment of immune cell subsets as well as determining immune checkpoint abundance on CD3^+^ (P2) and CD3^−^ (P8) cells. Lower panel: Phenotypic quantification. **d** Immunophenotyping of splenocytes from mice of all groups. Given are the percentage numbers of positive cells ± SD resulting from 20,000 events measured on a flow cytometer. *p < 0.05 vs. control; #*p* < 0.05 vs. lysate; §*p* < 0.05 vs. CPX + lysate; one-way ANOVA (Holm Sidak method)

Preconditioning with GEM or CPX prior to vaccination delayed tumorigenesis, with resulting prolonged survival (Fig. 3a). 33% of mice even remained tumor free until the experimental endpoint in the GEM vaccine group vs. only 11% in CPX pretreated mice (vs. 15.4% vaccination alone). Additional differences were seen in tumor distribution between these treatment groups. Mice pretreated with CPX before vaccination developed lymphomas more frequently than GIT (5/9 vs. 2/9 mice, respectively). Of note, lymphomagenesis was delayed (34.3 ± 6.9 weeks) compared to GEM + vaccination (23.5 ± 13.4 weeks) and vaccination alone (30.8 ± 10.0 weeks). Vice versa, GEM preconditioning resulted in more frequent gastrointestinal tumor formation (4/9 mice vs. lymphomas: 2/9 mice). Here again, tumorigenesis was slightly decelerated (44.6 ± 4.6 weeks vs. 42.0 ± 8.5 weeks vaccination alone) (Table 2).

**Table 2:** Effect on prophylactic chemo-immunotherapy on tumorigenesis and tumor spectrum in MLH1^−/−^ mice

Intervention	mean age of onset [weeks ± SD]		tumor type [%]			mice [%]
	Lymphoma	GIT	Lymphoma	GIT	other	tumor free
control^a^	25.3 ± 11.7	35.5 ± 9.3	62.1	34.7	3.2	0.0
lysate	30.2 ± 10.0	47.4 ± 8.5	51.7	33.3	0.0	15.0
GEM + vaccine	23.5 ± 13.4	53.4 ± 14.1	22.2	44.4	0.0	33.3
CPX + vaccine	34.3 ± 6.9	47.7 ± 5.9	55.5	33.3	0.0	11.1

^a^GEM and CPX treated mice without vaccine were grouped together, *GIT* gastrointestinal tumor

Single prophylactic application of GEM or CPX without subsequent vaccination did not influence tumor formation. All mice developed tumors within the expected time frame (Table 2). Consequently, data from control mice were pooled and the presented ones refer to these groups.

### Immune status and tumor microenvironment upon prophylactic chemo-immunotherapy

Prior to immune monitoring during prophylactic vaccination, the immune status of MLH1^−/−^ mice was examined in comparison to wildtype and heterozygous MLH1 mice (Fig. 3b). This analysis revealed marked differences in certain immune cell subsets, with a trend towards higher numbers of circulating CD11b^+^Gr1^+^ MDSC, CD200R^+^ monocytes - and immune-checkpoint-molecule positive cells – all of them known as inhibitory receptors with the capacity to down-modulate cellular activation [23, 24]. Of note, this imbalance between individual cellular subtypes was more evident in aged mice (≥32 weeks), indicative for a slightly impaired immune function in MLH1^−/−^ mice irrespective of tumor stage (Fig. 3b).

Subsequent immune monitoring (Fig. 3c, upper panel) during vaccination revealed increased relative numbers of CD3^+^CD4^+^ T helper and CD3^+^CD8^+^ cytotoxic T cells in mice pretreated with GEM or CPX (Fig. 3c lower panel). Immunological changes were evident until day 63 of vaccination, but declined afterwards (Fig. 3c). Assessment of MDSC revealed no significant changes between the individual treatment groups.

**Fig. 4:**
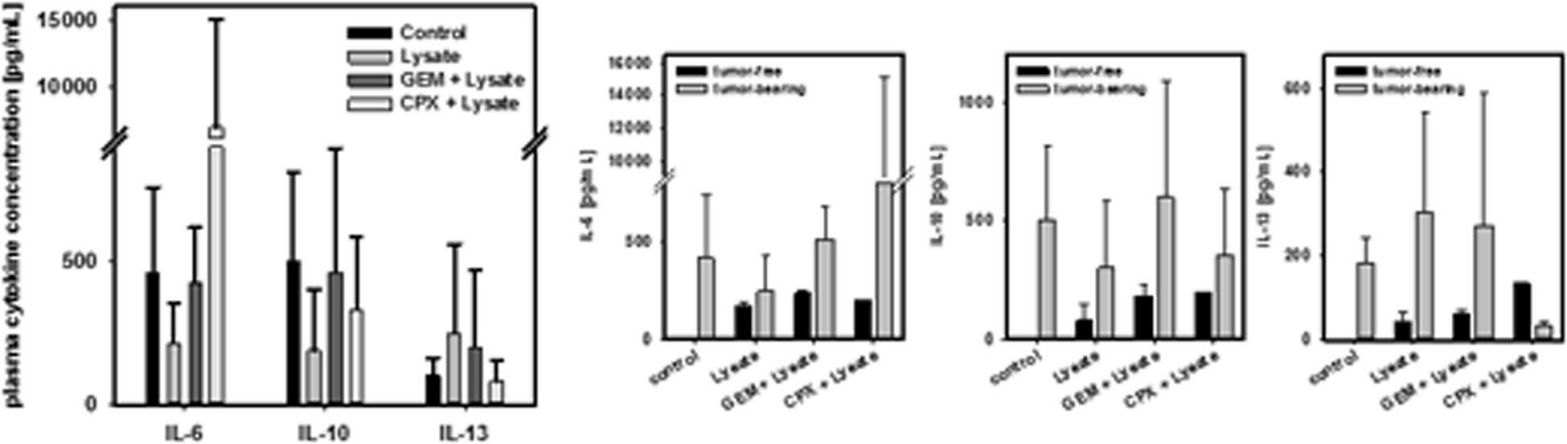
Plasma cytokine levels of IL-6, IL-10 and IL-13 from mice with prophylactic chemo-immunotherapy and controls (upper graph). Differences between tumor-free and tumor-bearing mice (lower graphs). Plasma samples were collected at the experimental endpoint and cytokine levels were determined as described in material and methods

Thereafter, spleens from vaccinated and control mice were analyzed with respect to immune cell subpopulations. Spleens from vaccinated mice with CPX or GEM pretreatment had higher relative numbers of T cells and a trend towards lower MDSC numbers (Fig. 3d). Likewise, percentages of Treg as well as LAG-3^+^ cells were lower in these groups and most evident in the GEM + vaccine group, accompanied by low amounts of IL-6, but higher levels of the Th2-cytokine IL-13 (Fig. 4). Of note and as expected, cytokine patterns differed between individuals depending on whether mice developed tumors or not (Fig. 4).

**Fig. 5:**
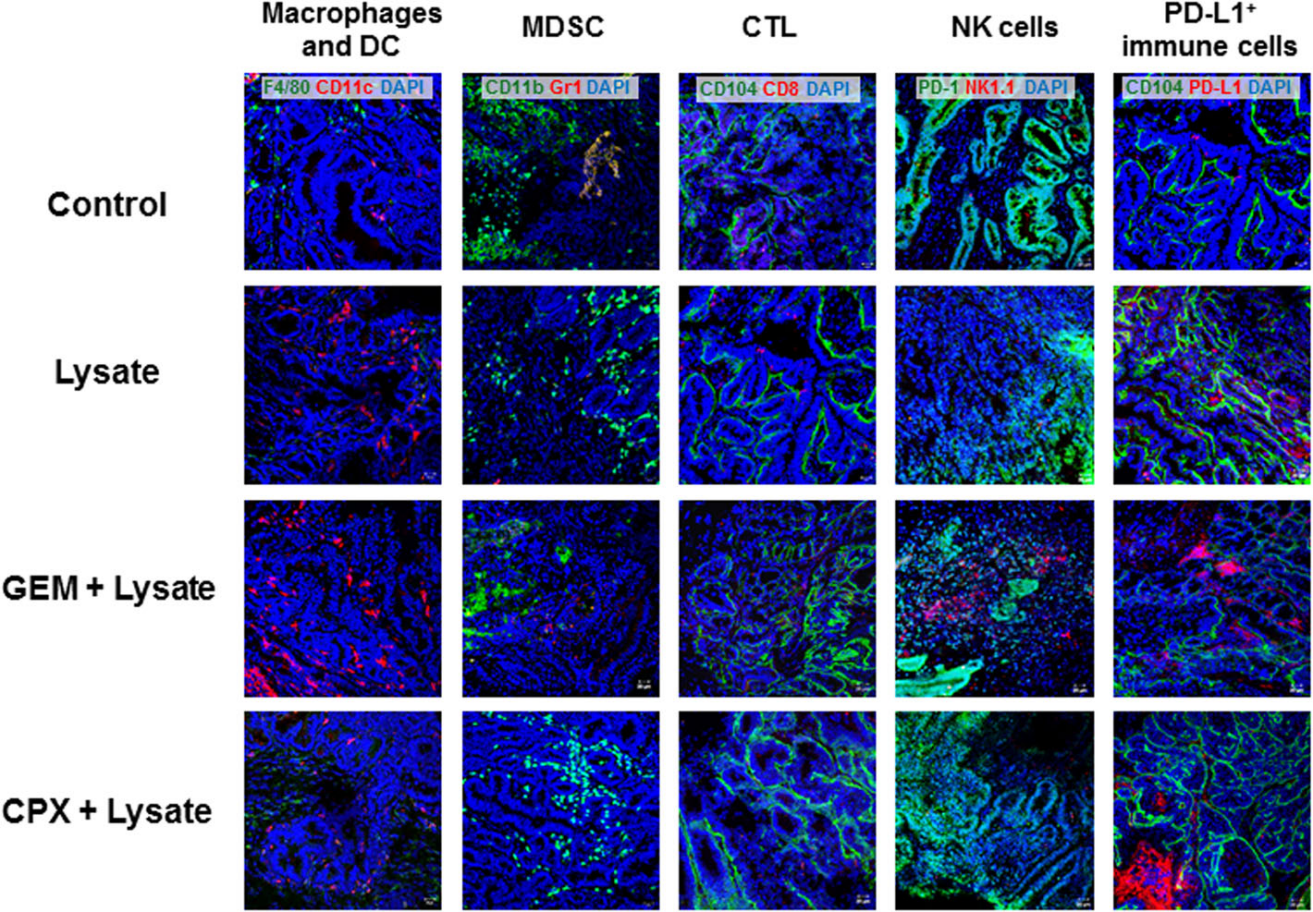
Representative micrographs of tumor microenvironment after prophylactic chemo-immunotherapy. GIT were resected from mice of all groups, cryopreserved and cut into 4 μm slides for immunofluorescence analysis. Upon blocking, slides were stained with fluorochrome-labeled monoclonal antibodies and DAPI for nuclear staining. Pictures were done on a confocal laser scanning microscope (Zeiss) using 20x objectives

Next, the tumor microenvironment was studied in detail. All vaccinated mice had higher numbers of infiltrating CD11c^+^ DC (Fig. 5). Mice preconditioned with GEM or CPX had additionally lower amounts of CD11b^+^ infiltrates and no MDSC in the tumor microenvironment. Numbers of tumor-infiltrating CTL increased only marginally in the combination. NK cells were interestingly higher in the GEM + vaccine group than in the CPX + vaccine group and almost absent in control and vaccinated tumors without pretreatment. Immune checkpoint molecule PD-L1 was highly upregulated on infiltrating cells in the MLH1^−/−^ tumor microenvironment.

### Therapeutic chemo-immunotherapy

**Fig. 6:**
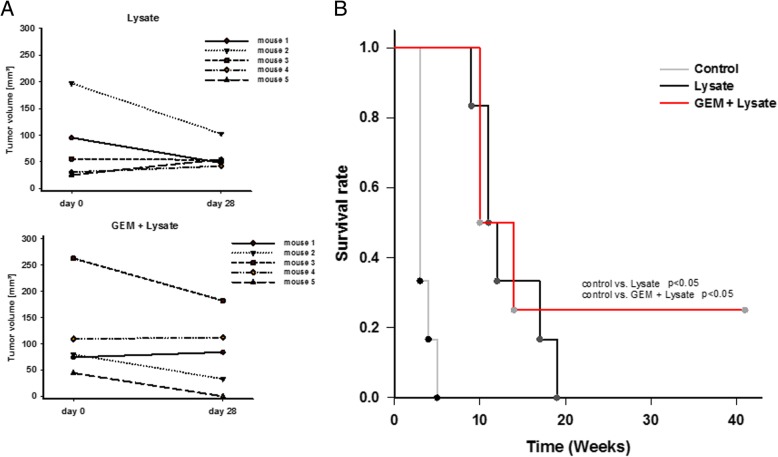
Tumor size and Kaplan-Meier survival curve during therapeutic chemo-immunotherapy. **a** Tumor volume in mm^3^ as determined by PET/CT imaging. Depicted are the mean tumor sizes at start of treatment and after 28 days of each individual MLH1^−/−^ mouse. **b** Log rank survival analysis of treated and control mice. Mice receiving chemo-immunotherapy were given GEM (100 mg/kg bw, i.p.) 24 h before vaccination (tumor lysate), followed by repetitive local applications of the vaccine (10 mg/kg bw, s.c., *n* = 5 mice). An additional group of mice received the vaccine (10 mg/kg bw, s.c., *n* = 5 mice). Control mice received single GEM (100 mg/kg bw, i.p., *n* = 3 mice) injection or were left untreated (*n* = 5 mice/group). Control vs. lysate *p* < 0.05; control vs. GEM + lysate p < 0.05

Next, MLH1^−/−^ mice with confirmed GIT were assigned to chemo-immunotherapy, based on the successful prevention of tumorigenesis by preconditioning with GEM. Tumor formation in the gastrointestinal tract was confirmed by in vivo imaging technique using ^18^F-FDG PET/CT. Mice developed 3.0 ± 1.7 tumor nodules in average (vs. vaccination alone: 3.5 ± 1.7 tumors) with a mean tumor volume of 110.1 ± 90.6 mm^3^ at start of treatment (vs. vaccination alone: 93.4 ± 74.8 mm^3^). GEM was given 24 h before vaccination, followed by repetitive local application of the vaccine. This regimen was well tolerated without having any serious side effects, like weight loss, anemia, or gastrointestinal disorders. Repeated in vivo imaging at day 28 or 35 of therapy revealed disease control which was, however, comparable to vaccination alone (26% growth reduction vs. vaccination alone: 31% growth reduction) (Fig. 6a). In one case, tumor nodules completely regressed and this mouse remained tumor free until the experimental endpoint (> 40 weeks) (Fig. 6a). Overall survival was quite similar between the two treatment arms, but significantly longer than control mice either given GEM once or left untreated (Fig. 6b).

**Fig. 7:**
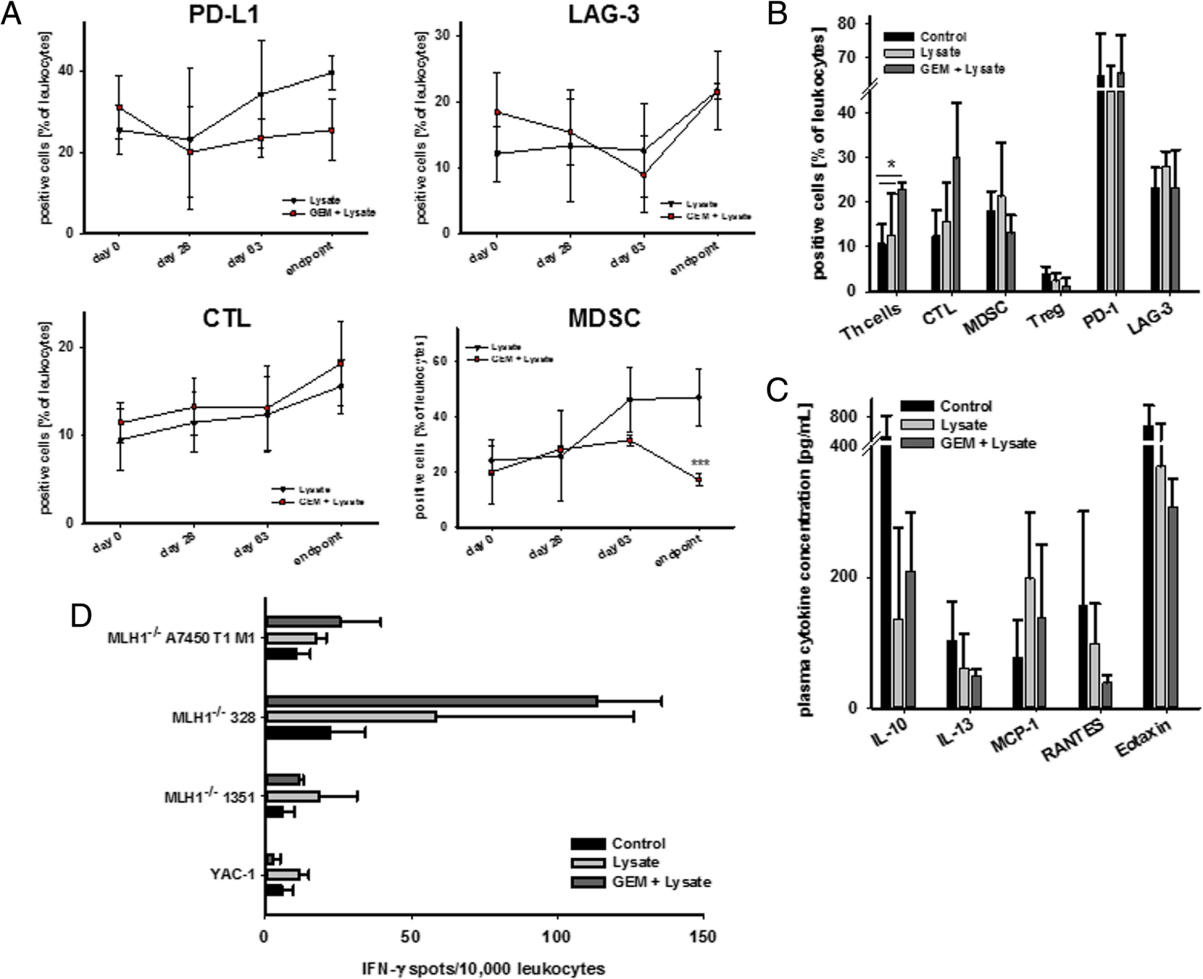
Immune monitoring, plasma level and IFN-γ ELISpot during and after therapeutic chemo-immunotherapy. **a** Flow cytometric immune monitoring of blood leukocytes was done routinely during vaccination and at the experimental endpoint. Given are the percentage numbers of positive cells ± SD resulting from 20,000 events measured on a flow cytometer. *** *p* < 0.001 vs. control; T-test. **b** Immunophenotyping of splenocytes from mice of all groups. Given are the percentage numbers of positive cells ± SD resulting from 20,000 events measured on a flow cytometer. * *p* < 0.05 vs. control; one-way ANOVA (Holm Sidak method). **c** Plasma cytokine levels of IL-10, IL-13, MCP-1, RANTES, and Eotaxin from mice with therapeutic chemo-immunotherapy and controls. Plasma samples were collected at the experimental endpoint and cytokine levels were determined as described in material and methods. **d** Number of IFN-γ secreting cells after over-night incubation of splenocytes (=effector cells) and tumor target cells (MLH1^−/−^ 7450 T1 M1, MLH1^−/−^ 328, MLH1^−/−^ 1351, and YAC-1). Lymphocytes were isolated from mice of all groups showing increased reactivity post treatment

Accompanying immune phenotyping revealed differences between treatment groups, with lower numbers of circulating MDSCs, PD-L1^+^ as well as LAG3^+^ immune cells in the GEM + vaccine group (Fig. 7a). NK cell numbers even remained low in this group of mice, while numbers of CD3^+^CD8^+^ cytotoxic T cells gradually increased. This altered immune phenotype was also detectable in spleens (Fig. 7b). Here again, T cell numbers (both Th and CTL) increased after chemo-immunotherapy, while immune regulatory cells were found to be low. Amounts of PD-1^+^ and LAG-3^+^ cells did not significantly change during vaccination either with or without GEM pretreatment (Fig. 7b). Likewise, plasma cytokine levels were comparable between both treatments, with a tendency towards reduced Th2-directed responses were comparing with controls (Fig. 7c).

Immunological changes contributed to differential responses in ELISpot IFNγ assays. Lymphocytes from GEM + vaccine mice specifically recognized MLH1^−/−^ target cells, with even higher numbers than lymphocytes from vaccinated mice without preconditioning (Fig. 7d). Along with the observed low amount of circulating NK cell numbers, recognition of NK cell target YAC-1 could be largely neglected.

### Tumor microenvironment

**Fig. 8:**
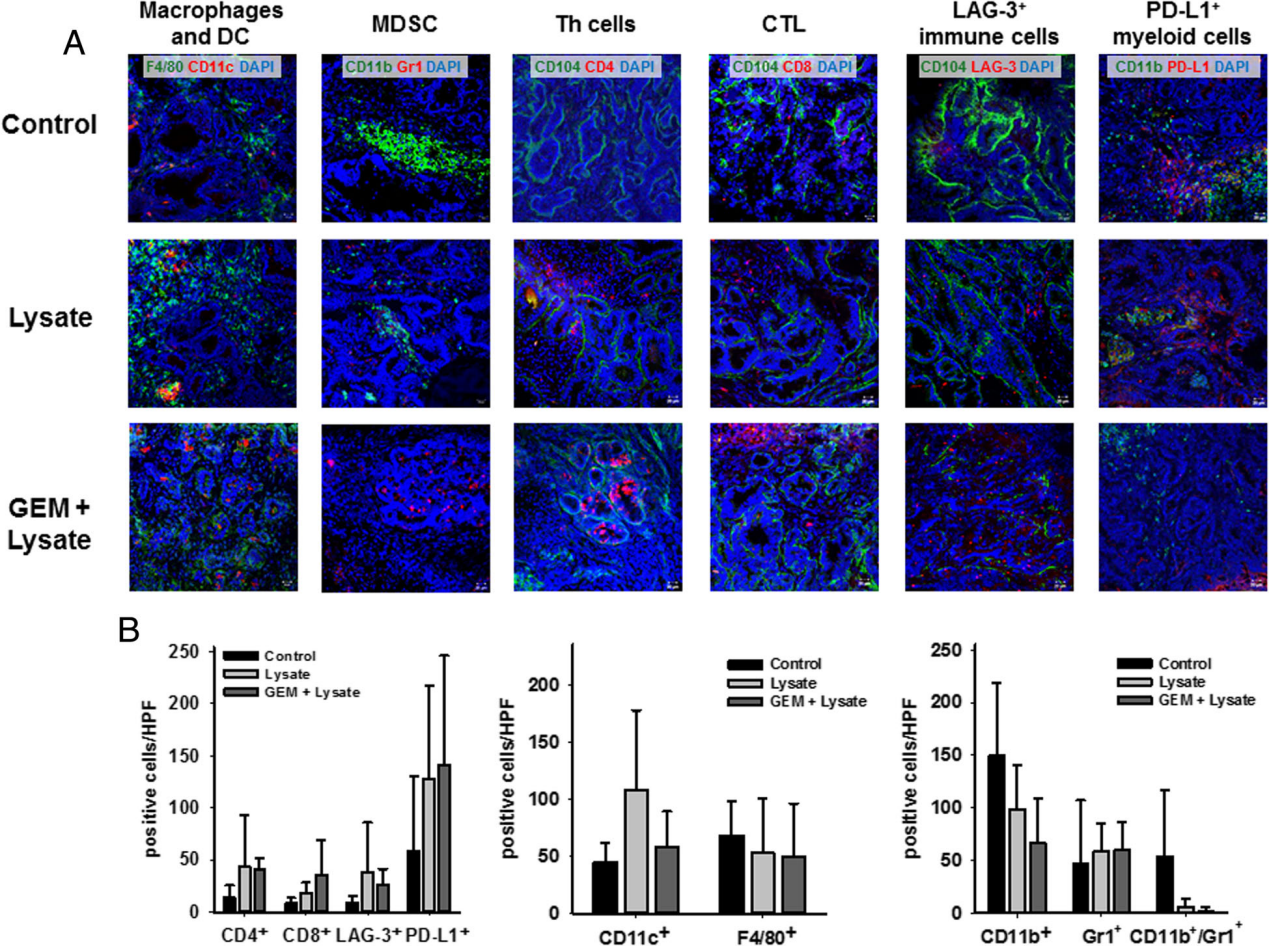
Tumor microenvironment after therapeutic chemo-immunotherapy. **a** GIT were resected from mice of all groups, cryopreserved and cut into 4 μm slides for immunofluorescence analysis. Upon blocking, slides were stained with fluorochrome-labeled monoclonal antibodies and DAPI for nuclear staining. Pictures were done on a confocal laser scanning microscope (Zeiss) using 20x objectives. **b** Quantification of immunofluorescence analysis. Data result from five individual fields/tissue (*n* = 3–5 mice/group). Values are given as mean ± SD

Detailed assessment of the tumor microenvironment from treated mice identified increased numbers of infiltrating CD4^+^ and CD8^+^ T cells in both treatment arms, but at higher levels upon chemo-immunotherapy (Fig. 8a, c). However, LAG-3 was also upregulated on infiltrating immune cells. With regard to other immune regulatory cellular infiltrates, differences were apparent between vaccinated mice and those treated with the combination (Fig. 8). Infiltrating CD11b^+^Gr1^+^ MDSC completely diminished in the GEM + vaccine group (2.0 ± 3.8 cells/HPF vs. vaccine: 6.3 ± 6.9 cells/HPF vs. control: 53.1 ± 63.1 cells/HPF), residual myeloid cells were mostly Gr1^+^CD11b^−^ granulocytes. CD11b^+^ myeloid cells, if any, had no expression of PD-L1 and may thus not be regarded as immunosuppressive response. In support of this finding, levels of CD11c^+^ DC increased upon (chemo-) immunotherapy (58.2 ± 31.1 cells/HPF vs. vaccine: 107.5 ± 70.1 cells/HPF vs. control: 45.0 ± 16.5 cells/HPF), while numbers of F4/80^+^ (tumor-associated) macrophages decreased (50.3 ± 46.1 cells/HPF vs. vaccine: 52.7 ± 48.2 cells/HPF vs. control: 68.3 ± 30.2 cells/HPF), indicative for phenotypic (and eventually functional) polarization (Fig. 8).

## Discussion

Recent years have seen much progress in understanding the interplay of chemotherapy and immunotherapy as well as in identifying drugs with immunomodulatory potential. While most agents failed to interfere positively with the immune system [22], GEM and CPX are two prominent candidates likely boosting vaccination and thus improving therapy. GEM assists in elimination of tumor-induced MDSC via apoptosis/necrosis and may even prevent MDSC maturation and activation [25]. GEM was also shown to support DC vaccination in murine pancreatic cancer models and in a phase I clinical study using WT1 peptide as vaccination antigen [26]. Besides, we and others were able to show that GEM exerts direct toxic effects towards MMR-D tumor cells of different origin (i.e. solid tumor vs. hematological malignancies) [27, 28]. Inhibition of cell proliferation was achieved at concentrations well below plasma levels under standard therapy [27] and accompanied by classical signs of ICD induction (exposure of CalR, HMGB1 release), the latter shown here on three low-passage murine MMR-D cell lines. CPX by itself has opposite effects: A single low dose of CPX given 1 to 3 days before vaccination conquers tumor-induced tolerance. However, CPX concurrently with or subsequently applied attenuates T cell immune responses through the PD-1-PD-L1 axis [29]. Successful combination of immune-stimulating vaccination and cytotoxic therapy depends on the choice of drug as well as its ability to induce ICD upon application in the right dose and treatment schedule. On a basis of these findings, we now focused on single chemotherapy prior to vaccination. MLH1^−/−^ mice, representing a valuable preclinical model for designing vaccine trials, were given chemotherapy once before vaccination, following recommendations of a recent study [30].

Prophylactic chemo-vaccination prolonged tumor-free time and accordingly overall survival. Of note, one third of mice exposed to GEM before vaccination remained completely tumor-free until the experimental endpoint. This effective tumor prevention was accompanied by increasing numbers of circulating T cells, lasting up to 2 month after start of treatment. Of note, vaccination alone or CPX preconditioning did not yield comparable results, again highlighting the potential of GEM as combinatorial agent for immunotherapy.

Another interesting finding of this study was the difference on type of tumor formation. Mice with CPX preconditioning gave primarily rise to lymphomagenesis and thus largely covered the expected tumor spectrum. Vice versa, chemo-immunotherapy with GEM reduced lymphomagenesis and also decelerated GIT formation. In this particular group however, lymphomas were early detectable, with one mouse showing pathological signs of an advanced malignancy 5 weeks after start of treatment. One may thus speculate that some premalignant, yet clinically not detectable, lymphatic cells were already present at vaccine initiation, finally contributing to treatment failure. Although proven to be effective in the in vitro cell culture system, single cycle chemotherapy with GEM was most likely not sufficient to inhibit cell growth in vivo. The aggressive nature of MMR-D lymphomas, making mice condemned to die within a short period of time, may additionally explain this result. Also this finding supports previous observations on only marginal entity-overlapping antitumoral capacity in the therapeutic situation [17]. Hence, vaccination against lymphomas is only effective in mice before evolving any oncogenic events. Molecularly, this can be attributed to the rapid and ongoing accumulation of novel (escape) mutations in MMR-D tumor cells [31–34]. Although the number of cases included here is far too low to make personalized recommendations, we would like to strengthen that prophylactic vaccines should be given early in life to maximize the potential to delay or even prevent tumor formation. These findings are in agreement with experiences from other vaccination approaches to impede Human *papillomavirus* (HPV)-related cancer [35]. In here, HPV vaccination is efficient and potentially lifesaving if administered to females naive or unexposed to vaccine HPV types [36, 37]. This might be of particular significance for patients diagnosed with CMMR-D, likely to benefit from immunoprevention – similar to the approaches based on regular use of aspirin or ibuprofen [38]. Along with intensified screening, outcome may be improved by earlier recognition of asymptomatic tumors that are better resectable and eventually curable [39]. Having in mind that carcinogenesis is accelerated in these patients early intervention is even more desirable.

The exact mechanism by which GEM in conjunction with the vaccine prevented tumor formation in some MLH1^−/−^ mice remains elusive. Still, interference with naturally immunosuppressive circulating as well as tissue-specific cells is feasible. In support of this, MDSC and Treg numbers were low in spleens of mice receiving this treatment arm – of note, even after several months. Vice versa, levels of splenic CTL were generally higher. This was, however, rather drug independent likely to constitute a positive spin-off with limited relevance for outcome.

Prophylactic chemo-vaccination additionally influenced the tumor microenvironment. MDSC polarization was accompanied by DC infiltration as part of the ongoing immune stimulation. Though not analyzed in detail in the current work activation of CTL is expected and may trigger a proinflammatory state [40]. Finally, these cells have the capacity to kill tumor cells directly [41]. Of note, a very recent single-arm, open-label phase I clinical trial confirmed the safety and efficacy of intratumorally injected activated DCs in patients with solid tumors [42]. In our study, immunosuppressive checkpoint molecules were upregulated in tumors of vaccinated mice to blunt tumor cell killing [43] and most likely explains final tumor formation in this immune-privileged microenvironment.

Overcoming the variety of immunosuppressive mechanisms pre-established in the tumor microenvironment is demanding for therapeutic approaches. Targeting therapy-induced suppressor cells may augment the long-term efficacy of vaccination. However, GEM application prior to vaccination had marginal impact on vaccine-induced tumor remission. This vaccine, providing a mixture of undefined antigens and likely to activate polyclonal immune responses, was effective in tumor cell killing by itself [44, 45]. Applying higher doses and/or more cycles of chemotherapy may improve outcome. Also, combinations of chemo-immunotherapy [46] and immune checkpoint inhibition are expected to move the field forward. Still, the complete tumor remission in one case is promising and warrants further investigations.

This approach sheds light on antitumoral mechanisms that include modulation/induction of MMR-D-specific immune responses as well as reshaping of the tumor microenvironment [47]. Hence, chemotherapy-induced cell death enhanced cross-priming, thereby increasing T-cell-driven responses either by recognizing tumor-derived neo-antigens (mutated proteins, re-activated antigens, etc.) or differentially expressed molecules (tumor-associated antigens) [48]. This was additionally proven on a functional level, in which syngeneic MLH1^−/−^ tumor targets evoked specific IFN-γ release of lymphocytes from vaccinated mice +/− GEM pretreatment. Of note, we observed only minor entity-overlapping responses (i.e. GIT vs. lymphoma) providing another evidence for differentially expressed target antigens among MLH1^−/−^ tumors. Of particular interest in this context is the question whether vaccination with a lymphoma lysate would yield comparable results. However, this has to be addressed prospectively.

Finally, immune interference is a complex phenomenon. The immunosuppressive nature of the tumor microenvironment is a key limitation to therapeutic vaccination. Breaking the tolerance by depleting or ‘re-educating’ immunosuppressive cell types remains the key to unleash antigen-specific immune responses. We here present a strategy to positively influence the choice between dominant immunosuppression versus inflammation, antigen cross-presentation, and epitope spreading that warrants further improvement.

## Conclusions

This study describes a strategy to delay MMR-D driven tumorigenesis. We were able to show that combined application of low-dose chemotherapy with immune-stimulating vaccination significantly prolongs survival both in a prophylactic and therapeutic setting. Depleting or ‘re-educating’ immunosuppressive cell types in conjunction with a cellular cancer vaccine is a promising concept for prospective vaccine-tailored immunotherapeutic trials.

## Abbreviations

5-FU: 5-Fluoruracil
CalR: Calreticulin
CPX: Cyclophosphamide
CTL: Cytotoxic T lymphocytes
DC: Dendritic cells
GEM: Gemcitabine
GIT: Gastrointestinal tumor
HMGB1: High-mobility group protein 1
HPF: High power field
HPV: Human *papillomavirus*
ICD: Immunogenic cell death
MDSC: Myeloid derived suppressor cells
MMR-D: Mismatch repair deficiency
PD: Programmed death
PD-L1: Programmed cell death *1* ligand *1*
Treg: Regulatory T cells
